# A Magnetic Bead-Based Sensor for the Quantification of Multiple Prostate Cancer Biomarkers

**DOI:** 10.1371/journal.pone.0139484

**Published:** 2015-09-30

**Authors:** Jesse V. Jokerst, Zuxiong Chen, Lingyun Xu, Rosalie Nolley, Edwin Chang, Breeana Mitchell, James D. Brooks, Sanjiv S. Gambhir

**Affiliations:** 1 Department of Radiology, Molecular Imaging Program at Stanford (MIPS), Stanford University, Stanford, California, United States of America; 2 Department of Urology, Stanford University, Stanford, California, United States of America; 3 Bioengineering, Materials Science & Engineering, Bio-X, Stanford University, Stanford, California, United States of America; University of Michigan, UNITED STATES

## Abstract

Novel biomarker assays and upgraded analytical tools are urgently needed to accurately discriminate benign prostatic hypertrophy (BPH) from prostate cancer (CaP). To address this unmet clinical need, we report a piezeoelectric/magnetic bead-based assay to quantitate prostate specific antigen (PSA; free and total), prostatic acid phosphatase, carbonic anhydrase 1 (CA1), osteonectin, IL-6 soluble receptor (IL-6sr), and spondin-2. We used the sensor to measure these seven proteins in serum samples from 120 benign prostate hypertrophy patients and 100 Gleason score 6 and 7 CaP using serum samples previously collected and banked. The results were analyzed with receiver operator characteristic curve analysis. There were significant differences between BPH and CaP patients in the PSA, CA1, and spondin-2 assays. The highest AUC discrimination was achieved with a spondin-2 OR free/total PSA operation—the area under the curve was 0.84 with a p value below 10^−6^. Some of these data seem to contradict previous reports and highlight the importance of sample selection and proper assay building in the development of biomarker measurement schemes. This bead-based system offers important advantages in assay building including low cost, high throughput, and rapid identification of an optimal matched antibody pair.

## Introduction

Prostate cancer (CaP) is the second leading cause of cancer death in US men, yet effective screening and prognostication tools have remained elusive due to non-specific assays and biomarkers [1]. While enzyme-linked immunosorbent assay (ELISA) is the gold standard for measuring serum proteins, it suffers from long assay development times for new biomarkers, high sample volumes, and requires many complicated operating steps. ELISA also can suffer from high background and non-specific interferences because it uses an optical-based reporting scheme.

To solve this, many competing technologies have been proposed including chemiluminescent-, microfluidic- [2], nanotechnology- [3], and magnetic-based [4] approaches. However, many of these approaches suffer from high cost, low throughput, long assay construction times, and the need for a skilled operator. Thus, a powerful but simple assay platform with universal utility for a broad variety of protein biomarkers has yet to be reported. In particular, the biomarker community would be well served by analytical tools for panels of biomarkers.

Indeed, several recent studies have suggested that panels of biomarkers may be more effective at the early identification of cancer and classification of disease aggressiveness than the prostate specific antigen [5] or other single point assays [6, 7]. Indeed, due to the deep biological complexity of cancer, it does seem logical that assaying for more than one biomarker would be beneficial in accurately prognosticating or detecting disease in the highest number of patients. These panels include transmembrane proteins [8], metabolites [9], auto-antibodies [10], non-coding RNA such as *PCA3* [11], gene fusions including *TMPRSS2-ERG* [12], circulating tumor cells [13, 14], DNA methylation patterns [15], gene profiling [16], exosomes/prostasomes [17], and many others [18].

Here, we describe a piezoelectric membrane-based approach that uses specific interactions with magnetic beads to quantify prostate cancer biomarkers with many advantages for assay building: 1) This approach is nearly matrix-free because it uses magnetic- and piezo-based quantification schemes rather than optics; 2) The system can rapidly (<2 days) identify a matched antibody pair for immunoassay; 3) The system has detection limits log orders lower than ELISA because of the avidity of the three dimensional bead versus planar ELISA; and 4) The system is largely operator-independent.

We used this tool to create a prostate cancer biomarker panel and evaluated its utility from samples from CaP patients and patients with benign prostatic hypertrophy (BPH). We focused on serum proteins due their ease of sampling, established role in disease identification and monitoring, and the large number of banked serum samples available for testing. We included newly identified markers as well as established PSA-based tests. Seven proteins were included in this panel based on the current literature understanding of CaP:

- PSA [5] is an enzymatic glycoprotein produced by the prostate with function in sperm motility [7]. This panel includes both free PSA (fPSA) uncomplexed to chaperone carbohydrate proteins and total PSA (tPSA) that includes both complexed and uncomplexed isomers [19, 20].
- Prostatic acid phosphatase (PAP) is a 100 kD glycoprotein synthesized in the prostate that hydrolyzes phosphate esters at acidic pH. PAP is documented in the historical literature and was used prior to the identification of PSA to detect and monitor CaP [21]. Panels utilizing PAP have shown increased specificity, but no increase in sensitivity [22].
- Carbonic anhydrase 1 (CA1) is a metalloenzyme implicated in the interconversion of dissolved CO_2_ and water into bicarbonate and is responsible for pH regulation. CA1 has recently been shown to be up-regulated in CaP patients through mass spectrometric profiling [23].
- Secreted protein acidic and rich in cysteine (SPARC), also known as osteonectin or basement-membrane protein 40 is a 40 kD protein implicated in the migration of prostate cancer cells [24] and metastatic prostate cancer [25].
- Interleukin-6 (IL-6) is an inflammatory cytokine. IL-6 and its ligand IL-6 soluble receptor (IL-6sr) are correlated with aggressive disease, including higher Gleason score, advanced stage, development of metastasis and decreased survival [26, 27]. Circulating levels of both IL-6 and IL-6sr are thought to result from the primary tumor as opposed to metastatic deposits [7].
- Spondin-2 (SPON2) works with the Wnt pathway to activate beta catenin and may facilitate morphogenesis [28]. SPON2 has been shown to be overexpressed in tissue culture media of androgen receptor positive prostate cancer cell lines [29, 30]. SPON2 serum tests were also shown to be elevated in patients with CaP relative to healthy controls [31].

This magnetic bead-based system correlates the concentration of biomarker to changes in acoustic frequency of a piezo-electric membrane [32]. The linear dynamic range of the assay was tuned to match the relevant concentration of these biomarkers in diluted serum [33]. We first identified matched antibody pairs and then characterized the assays for reproducibility, analytical sensitivity, and matrix interferences. We finally examined the clinical sensitivity and specificity of the panel with a retrospective trial of 240 banked human serum samples. To the best of our knowledge, this is the first example of these proteins integrated into a single panel and the most detailed use of this bead-based piezoelectric analysis system with important implications in CaP screening, monitoring, and treatment.

## Materials and Methods

### Antibodies

Unless otherwise stated, immunoreagents were functionalized in our lab and validated with either a recombinant or patient-purified protein standards. Optimal matched pairs including capture antibody (c.Ab) and detecting antibody (d.Ab) were obtained from the following vendors:

- **tPSA:** The c.Ab (Meridian p/n M66280M) the d.Ab (Meridian p/n M66276M) were both mouse monoclonal IgG.
- **fPSA:** The c.Ab (Meridian p/n M86806M) the d.Ab (Meridian p/n M66276M) were both mouse monoclonal IgG. For both tPSA and fPSA we used native human PSA prepared in our laboratory as the standard [34].
- **PAP**: The c.Ab (CosmoBio p/n SIM-2ZHCMP2-EX; clone Hyb-7432, described below as Cos2) was a mouse monoclonal, and the d.Ab (CosmoBio p/n SIM-2ZHCMP1-EX, described below as Cos1) was a mouse monoclonal. A recombinant protein standard (Fitzgerald p/n 30C-CP1016x) validated the assay. Other antibodies evaluated include a mouse monoclonal IgG1 clone 690017 from R&D Systems (RDMo) and a sheep polyclonal IgG p/n AF6249 from R&D Systems (RDPo).
- **SPARC:** The c.Ab (Invitrogen; p/n 33–5500 (On1-1)) was a mouse monoclonal, and the d.Ab (R&D Systems; p/n AF941) was a goal polyclonal. A recombinant protein standard (R&D Systems p/n 941-SP-050) validated the assay.
- **CA1**: The c.Ab (Abnova; p/n H00000759-M21) was a rabbit affinity-purified polyclonal, and the d.Ab (Abnova; p/n H00000759-D01P) was mouse monoclonal anti-CA1, IgG2b Kappa. A recombinant protein standard (Abnova p/n H00000759-P01) validated the assay.
- **IL6-sr:** The c.Ab (R&D Systems p/n AF227) was a goat polyclonal, and the d.Ab (R&D Systems p/n MAB227) was a mouse monoclonal. A recombinant protein standard validated the assay.
- **SPON2**: The c.Ab (R&D Systems p/n AF2609) was a goat polyclonal, and the d.Ab (Abnova p/n H00010417-MO1J) was a mouse monoclonal. A recombinant protein standard (R&D Systems) validated the assay.

### Assay Reagents

Magnetic beads and d.Abs were prepared as described previously [35]. Briefly, beads (1 mg, Bioscale, Inc.) were stored in anhydrous dimethylformamide and were collected by centrifugation and washed with PBS. After the last wash, we added PBS, 146 μL 4.1 M ammonium sulfate, and 5–20 μg of antibody. The amount of PBS was adjusted such that total volume was 300 μL. The solution was incubated overnight on a tube rotator turning at 4 rpm. The next day, the solution was washed four times with 0.1% Tween in PBS (PBST) and finally stored in PBS with 1% BSA and 0.01% sodium azide. Beads were stable for over 4 months.

The d.Abs were labeled with a NHS-fluorescein reagent (Pierce). The dye was added at a 20-fold molar ratio and incubated at room temperature in PBS at room temperature while protected from light. The conjugate was purified with spin columns and characterized with absorption spectroscopy to determine the final concentration and labeling efficiency.

### Analyzer and Assay Conditions

We used a commercially available piezo-electric analyzer for the protein measurements (VIBE, Bioscale, Inc.; see S1 Video). This system consists of a bench top analyzer and disposable reagent cartridge capable of analyzing 288 specimens and standards per run. The analyzer has embedded fluid handling, waste disposal, and data handling capabilities [33, 35]. Signal is reported in relative units via embedded software and is proportional to the number of beads immobilized on the membrane and thus analyte concentration. Hardware control and data analysis was performed with Bioscale commercial software (Vibe version 0.7.3).

**Fig 1:**
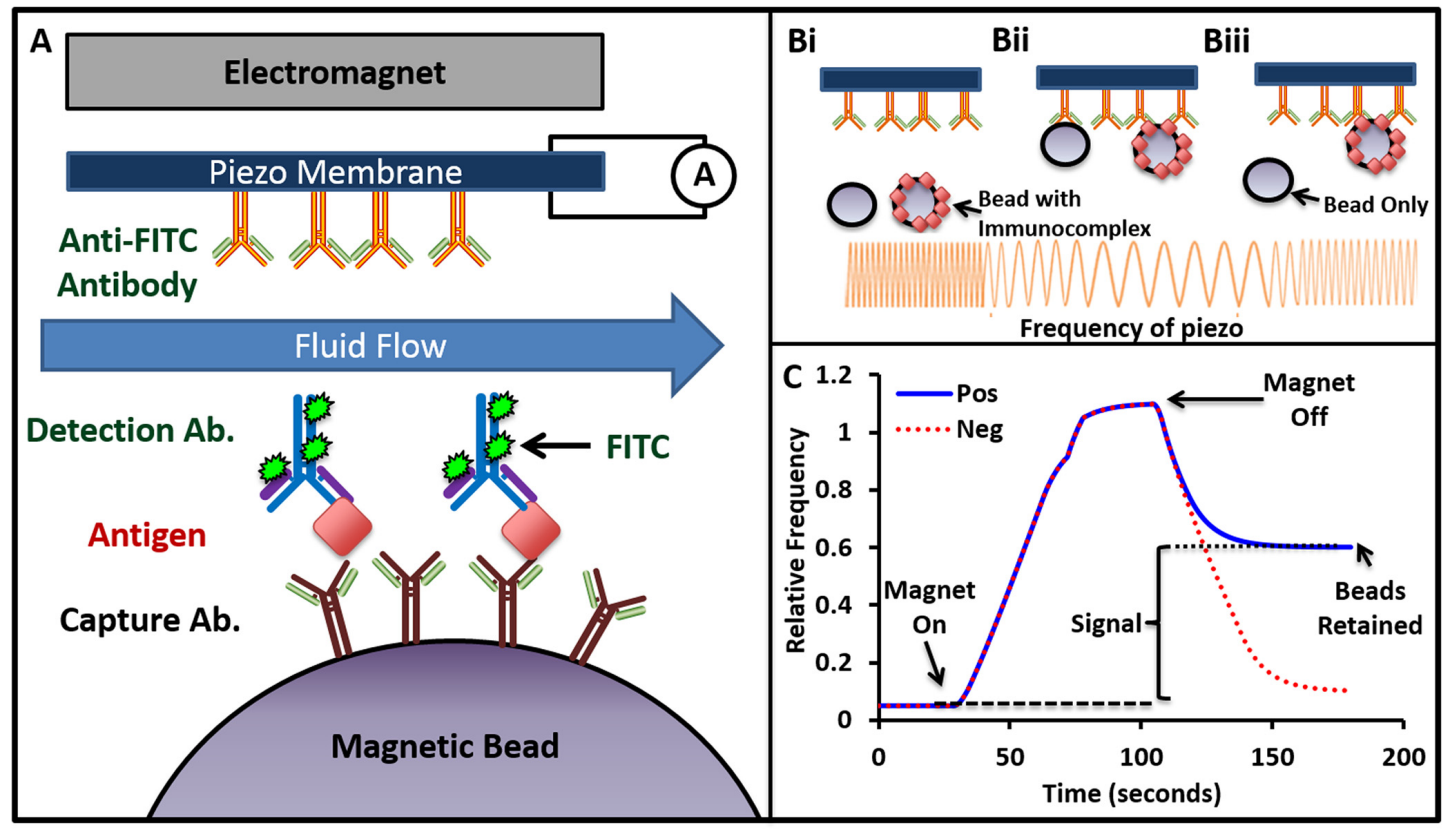
Detection Scheme. A. The c.Ab. is covalently bound to a magnetic bead that is incubated with sample and fluorescein-tagged d.Ab. This sample is introduced into a flow cell capped with a piezo-electric membrane coated with an anti-fluorescein antibody. An electromagnet above the membrane is controlled with embedded software. B. Upon magnetic perturbation, all beads (black circles) move towards the membrane (Bi), and beads with a completed immunocomplex (black circles coated with red dots) bind to the anti-fluorescein antibody (Bii). After the field is removed and flow restored, only beads with a completed immunocomplex remain bound (Biii). These beads alter the oscillation of the membrane (represented as orange sinusoidal curve), which is interpreted as signal in arbitrary units [36]. See S1 Video. In B, the solid blue line labeled Pos refers to beads in the presence of antigen, and the dotted red line labeled Neg refers to beads in the absence of antigen.

This system consists of a disposable 8-channel cartridge connected to fluidic handling robotics and an electrically controlled magnet. Samples and standards were prepared at 1:10–1:1000 dilution factor and plated into 96 well plates along with beads and fluorescent antibody. The specimens were prepared by mixing 80 μL of sample or standard with 20 μL of beads coated with c.Ab (900,000 beads/mL) and 20 μL of fluorescein-labeled d.Ab (1200 ng/mL). These reagents form an immunocomplex sandwich in the presence of the biomarker antigen (Fig 1A).

Up to 100 μL of this mixture was required for magnetic sorting and analysis. This mixture was incubated with shaking for 4 hours. The solution in each column of the plate (all 8 wells) was then auto-pipetted into individual flow channels within the 8-channel cartridge. After the solution was loaded into the chamber, a magnetic field was applied and all beads were drawn towards the anti-fluorescein IgG-coated piezo membrane in front of the magnetic field. Flow and dilution factors were adjusted such that the number of immobilized beads was between 2000–3000.

As these beads come into contact with this piezo membrane, the oscillation frequency is altered and recorded (Fig 1B). The magnetic field was maintained to allow interactions between beads with complete immunocomplexes and the membrane—the fluorescein tag on the d.Ab facilitated binding of the immunocomplex to an anti-fluorescein IgG on the piezo membrane. The field was then discontinued and flow resumed to remove non-specifically bound beads. The change in piezo frequency was then recorded as a measure of the number of bound beads and thus antigen concentration that completes the immunocomplex (Fig 1B).

### Samples

Banked serum samples had been collected over 20 years with informed consent by the Urology Department at Stanford University. We used serum because it has clotting factors removed, which can produce non-specific signal and background in plasma protein assays. Diagnosis was confirmed with 10- or 12-core biopsies at the time of sample collection. The Internal Review Board of Stanford University approved this study and the design of the informed consent. All participants provided written informed consent at the time of sample collection, and participant consent forms were curated along with the specimens at the Stanford Department of Urology. These samples were thawed, de-identified, diluted in PBS, and aliquoted for testing. We studied 240 samples selected at random from a bank of ~2,200 specimens curated at Stanford Radiology. The average age of the BPH controls was 65.9 ± 9.2 (30–87), and the average age for the CaP patients was 62.6 ± 6.4 (46–77); there was a statistically significant age difference between the two groups (p = 0.002). We have included this information in the text. There were 120 BPH samples and 100 cases of confirmed prostate cancer selected; all samples had PSA values between 2–20 ng/mL. Of the CaP samples, 32 were Gleason score 6 and 68 were Gleason score 7. As a control, there were also 20 samples from men with CaP collected after radical prostatectomy. Dilution factors were optimized empirically to give a spike recovery of 100% ± 10%. Pooled normal female serum was purchased from Atlanta Biologicals.

### Data Interpretation and Statistics

Signal units of the unknowns were converted into concentration units using a standard sigmoidal curve with Bioscale software (Vibe version 0.7.3). The detection range was defined as those calibration points that could be discriminated from their next nearest calibration points. Reference ranges were taken from clinical guidelines or the literature depending on the status of the biomarkers. We did a 2-tailed Student’s t test to compare the results of BPH and CaP samples in Excel 2010. The AUC and ROC curves and associated statistics were prepared with GraphPad Prism 6.04.

## Results

### Identification of Matched Pair

**Fig 2:**
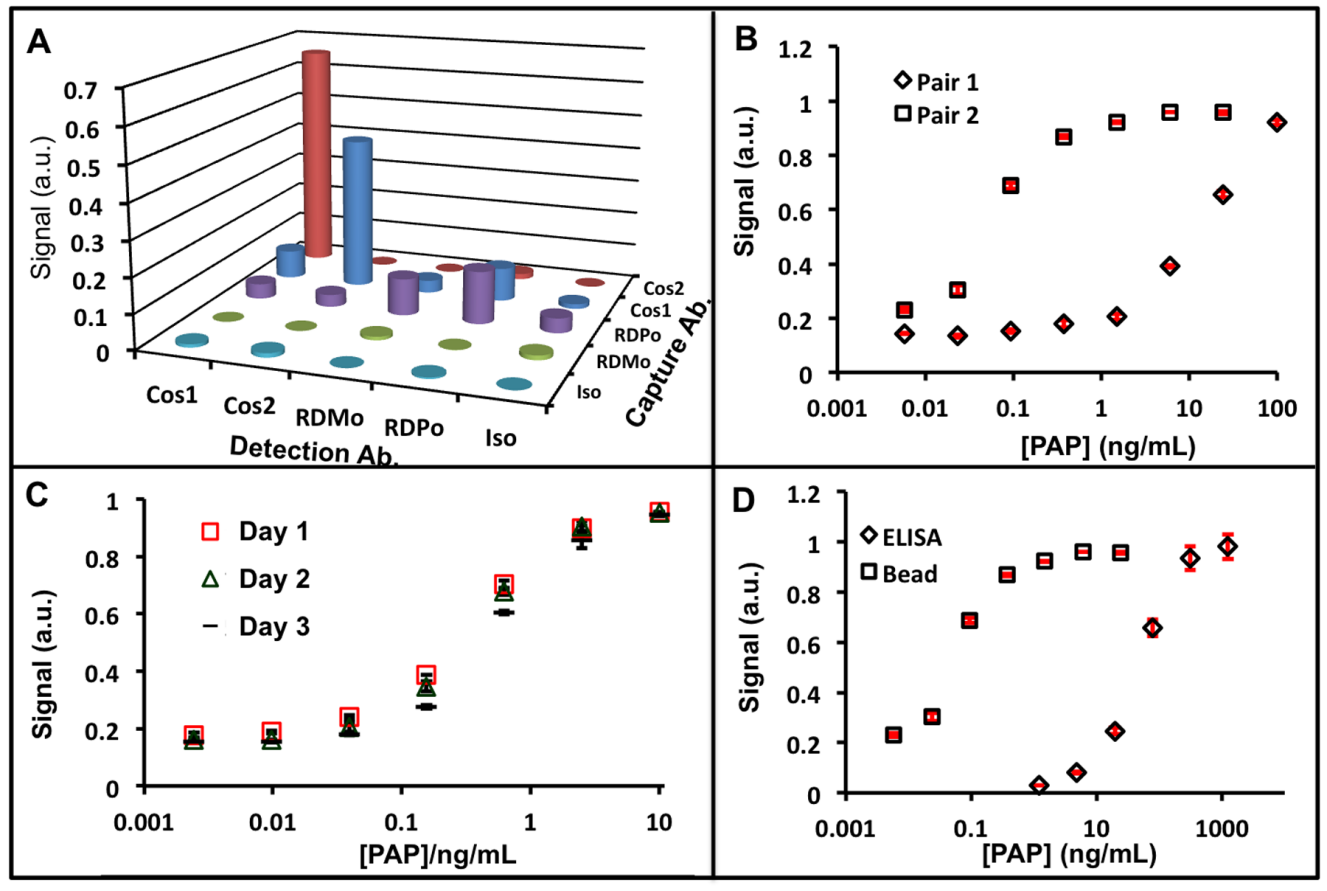
Assay Construction. **A)** Optimal antibody pairs were identified with a “checkerboard” assay that evaluated different antibody pairs as well as isotype controls. Metric plotted here is signal difference between the negative control and 1 ng/mL recombinant PAP. The different antibodies are defined in the Materials and Methods section. **B)** A full calibration curve illustrates the different sensitivities of different antibody pairs for PAP. Pair 1: Cos2 c.Ab.; Cos1 d.Ab. Pair 2: RDPo c.Ab.; RDMo. d.Ab. The red error bars represent the standard deviation of at least three replicate measurements. **C)** Reproducibility from day to day is <8%. **D)** The piezo-based approach shows 3 log orders improvement in analytical sensitivity versus direct ELISA when identical antibody pairs are used (Cos1/Cos2).

We first had to identify an optimal antibody pair, and the following section describes this approach using PAP as an example—results for other assays are presented in Table 1. Fig 2 shows representative data of how this was achieved for the PAP biomarker with a so-called “checkerboard assay.” Here, four different PAP antibodies as well as an isotype control were used as both the c.Ab and d.Ab. All four antibodies and an isotype control was used at antigen concentrations of 0 and 1 ng/mL. Fig 2A plots the signal difference between these two concentrations. For PAP the highest signal was with the Cos1 d.Ab and Cos2 c.Ab with a signal of 0.65 a.u. (Fig 2A). Inverting the order, i.e. Cos2 d.Ab and Cos1 c.Ab had a value of 0.43 a.u. The pair from R&D Systems had lower signal, and the isotope control antibody had signal <0.01 a.u. Thus, Cos1 and Cos2 were used for the remainder of the experiments.

**Table 1:** Optimized assay parameters. The best-matched pair with resulting dynamic range, reference range, and dilution factors are shown for the 7 biomarker assays as well as representative intra-assay variation values.

Biomarker	# Ab. Tested	Detection Ab.	Capture Ab.	Range (ng/mL)	Reference (ng/mL)	Dilution Factor	%CV
tPSA	4	M66276M (Meridian)	M66280M (Meridian)	0.04–50	< 4	1:5	0.69
Free/Total PSA	4	M66276M (Meridian)	M86806M (Meridian)	0.01–10	5–30% of tPSA	1:5	0.80
PAP	4	SIM-2ZHCMP1-EX (CosmoBio)	SIM-2ZHCMP2-EX (CosmoBio)	0.01–10	<2.1	1:20	0.51
SPARC	3	AF941 (R&D)	33–5500 (Invitrogen)	5–4000	<2000	1:20	0.51
CA1	4	H00000759 D01P (Abnova)	H00000759 M21 (Abnova)	0.1–50	<1000	1:300	0.55
IL-6sr	4	MAB227 (R&D)	AF227 (R&D)	0.15–10	<25	1:50	0.57
SPON2	2	H00010417-M01J (Abnova)	AF2609 (R&D)	0.38–390	n/a	1:5	0.76

Of course, changing the antibody pair changes the sensitivity of the curve. Fig 2B illustrates that by changing the c.Ab to RDPo and the d.Ab to RDMo, the sensitivity range changed from 0.01–1.0 ng/mL to 1 to 100 ng/mL. Finally, we compared the response curves of PAP via the bead-based system with ELISA using the same Cos1/Cos2 pair (Fig 2D). We show a 3-log order improvement in analytical sensitivity using the bead-based approach that highlights the enhanced analytical sensitivity due to avidity of the bead versus the planar ELISA substrate.

The amount of c.Ab and d.Ab per assay was also investigated (S1 Fig). The manufacturer recommended a bead working concentration of 150,000 beads/mL and 200 ng/mL d.Ab. We tested d.Ab values of 100 and 400 ng/mL as well as 10,000–300,000 beads/mL, but did not find any enhancement in dose-dependent curve from 10.0 to 0.015 ng/mL. At 1 ng/mL of recombinant standard PAP, the highest signal was achieved with 10,000 beads/mL and 100 ng/mL of d.Ab. However, these conditions also resulted in higher background signal at lower concentrations (S1 Fig). Thus, 150,000 beads/mL and 200 ng/mL d.Ab were used for all subsequent assays.

### Assay Characterization

We next examined both intra-assay (between wells run on the same day at the same time) and inter-assay variance (difference between the average of runs on different days; Fig 2C). For PAP, intra-assay variance ranged from 0.05% relative standard deviation [37] at 6 ng/mL to 5.47% at 0.02 ng/mL. The inter-assay variance on three different days was 7.1% RSD. All other assays had <7% variance for intra-assay variance and <10% for inter-assay variance. When making day-to-day comparisons it was critically important that the calibration curves be arranged in the 96-well plates the same way on each subsequent day. Because each row in the 96 well plate takes ~5 minutes to analyze, altering the location of the beads allows for longer incubation times, which can result in higher bead/immunocomplex accumulation and thus higher signal.

**Fig 3:**
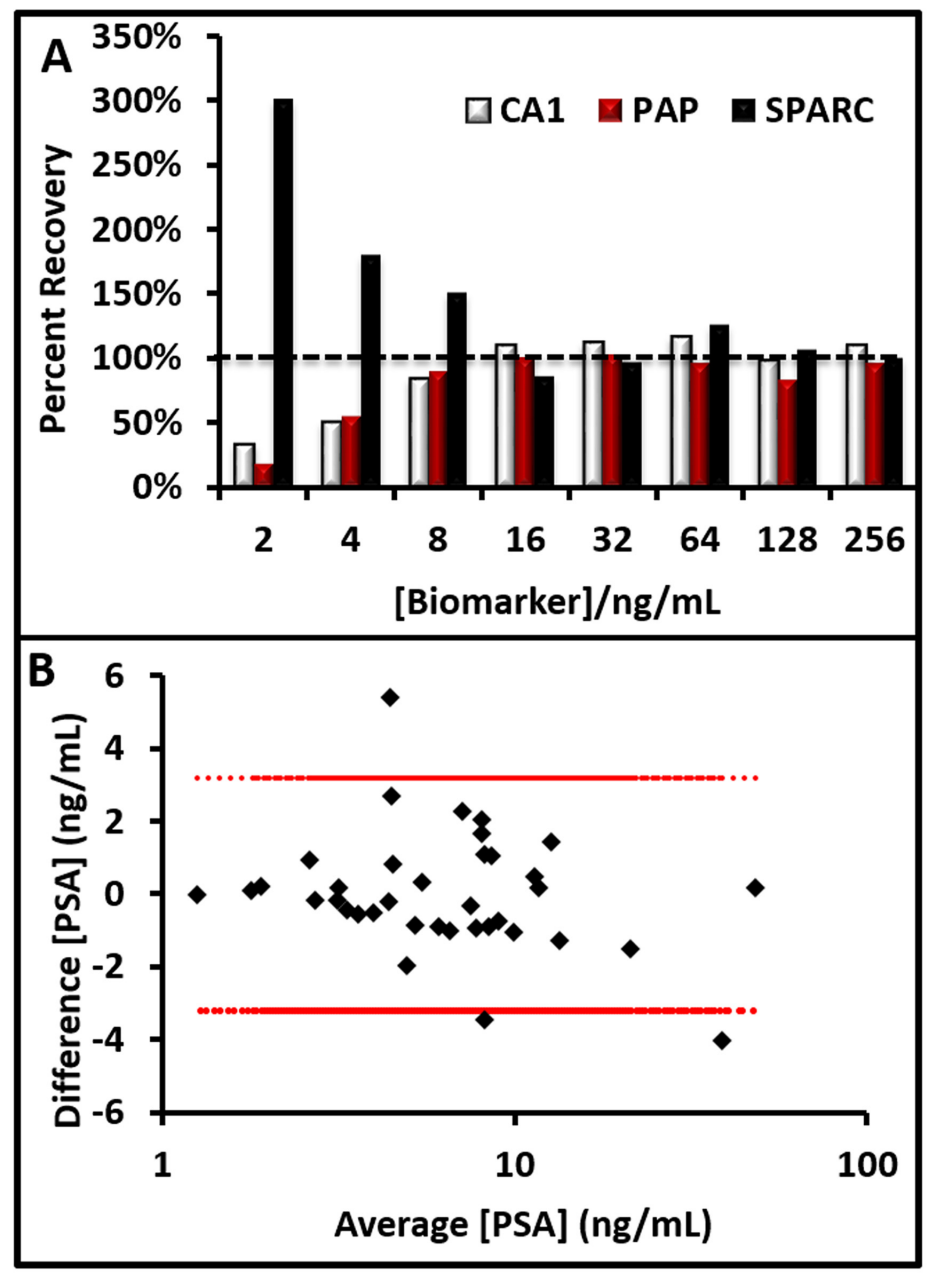
Serum Validation. **A)** Percent recovery for three representative biomarkers. Matrix effects can either dampen signal (CA1, PAP) or inflate signal (SPARC). Ideal dilution factors were dependent on sensitivity of the assay and reference range of the biomarker (Table 1). Black dashed line indicates 100% spike recovery. **B)** Bland-Altman plot [38] validating specimen integrity of a subset (N = 35) of the clinical samples with high sample volumes. Red dashed line indicates 95% confidence interval.

With an optimized matched antibody pair in hand, we next uncovered and corrected any matrix interferences again illustrating with PAP. We used pooled normal female serum that should contain no PAP. Serum at dilution factors from 1:2–1:256 was prepared and analyzed. Another batch of diluted samples was spiked with 0.5 ng/mL PAP. The samples were analyzed and the percent recovery plotted in Fig 3A as well as spike recovery data for SPARC spiking at 125 ng/mL and CA1 spiking at 10 ng/mL. The results showed that 100% recovery was achieved with 16-fold dilution factors for these analytes. Decreased and increased spike recovery values are due to non-specific binding of other serum proteins to the antibodies. The other five biomarkers were optimized similarly.

### Clinical Samples

The first step was to validate sample stability. To do this, we reanalyzed the PSA values of a subset (N = 35) of the samples that had high volumes of serum using a Beckman Access clinical analyzer (Hybritech) at the Stanford University Medical Center and compared this to the values originally measured at the time of collection. We created a Bland-Altman plot and showed that 92% of the samples were within the 95% confidence interval (Fig 3B). This validation only used those samples that had enough volume for both the clinical system and bead-based assay. The results indicated that the PSA integrity was maintained during storage.

**Fig 4:**
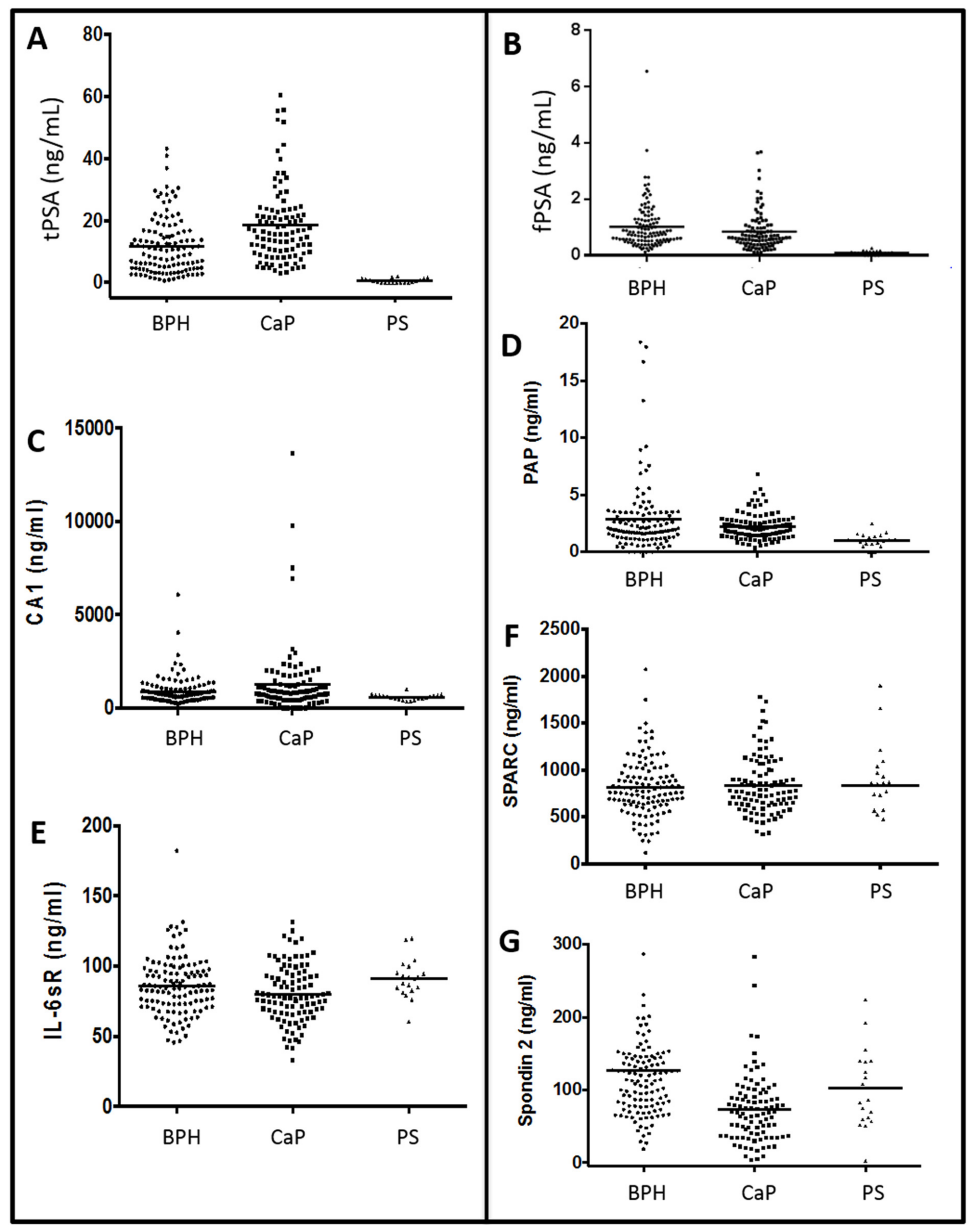
Serum Data. Raw values with means for BPH, CaP, and post-surgery (PS) samples. Biomarkers include tPSA (A), fPSA(B), CA1 (C), PAP (D), IL6-sr (E), SPARC (F), and SPON2 (G). Horizontal lines indicate mean values.

Next, we measured biomarker levels of all analytes in serum diluted to the concentrations shown in Table 1. The data for the BPH, CaP, and post-surgery CaP samples are shown in Fig 4. The average value from CaP patient samples are 1.5-, 1.6-, 0.83-, 0.94-, 0.79, and 1.03-fold higher than BPH for the CA1, PSA, IL6-sr, PAP, and SPARC assays, respectively. Tests with a significant difference between BPH and CaP were PSA (p<0.0001), CA1 (p = 0.03), SPARC (p = 0.049), and SPON2 (p<0.10^−6^).

**Fig 5:**
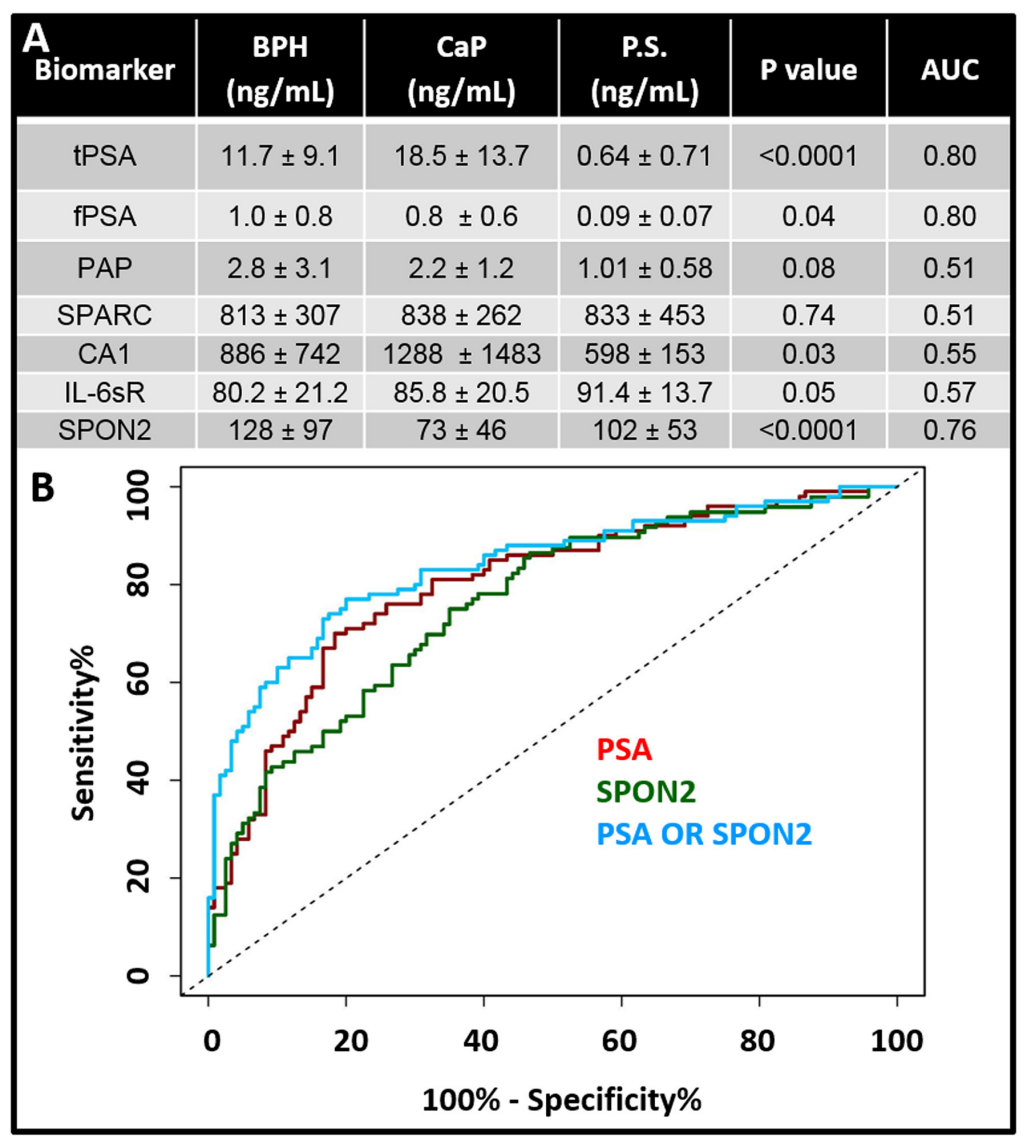
Clinical Data Analysis. A) Mean values with standard deviation for the seven biomarkers with BPH, CaP, and post-surgery [39] samples. Units are ng/mL. The p value reported here is the significance between the BPH and CaP samples. AUC values are also given and report discrimination between CaP and BPH. Lower panel presents ROC curves for PSA, SPON2, and PSA OR SPON2. The OR operator increases the AUC to 0.84 from 0.80 for PSA alone.

We created ROC curves and measured the AUC for all biomarkers using the biopsy-confirmed diagnosis and measured values (Fig 5). The PAP AUC was 0.51; 95% confidence interval 0.43–0.58 with p>0.50. The SPARC AUC was 0.51; 95% confidence interval 0.43 to 0.59 with p>0.50. The CA1 AUC was 0.55; 95% confidence interval 0.47 to 0.63 with p = 0.17. The IL-6sr AUC was 0.57; 95% confidence interval 0.50 to 0.65 with p = 0.06. The SPON2 AUC was 0.76; 95% confidence interval 0.69 to 0.82 with p<0.0001.

The AUC value for PSA is typical of literature values [40–42]. In one meta-analysis using free and total PSA, the aggregate for 41 studies with 19,643 participants was an AUC of 0.70 [41]. Unfortunately, other biomarkers did not improve the AUC with the exception of SPON2. That marker had an AUC value of 0.76 compared to the PSA AUC of 0.80. When the two were used in an OR operation, the AUC increased slightly 0.84. No other operations or other combinations of biomarkers increased the AUC curve.

We also studied whether any biomarkers could discriminate between Gleason 6 and Gleason 7 patients (S1 Table). None of the novel proteins showed a p value <0.05. While this dataset did show a very significant difference for tPSA (<0.01), this is likely due to the presence of some outlier values—the p value was 0.06 when we used the original clinical PSA values. We also did ROC analysis for different Gleason scores versus BPH and noted significance with IL-6sr. In comparing Gleason 7 patients versus BPH with IL-6sr, we found an AUC value of 0.66; the 95% confidence interval was 0.57 to 0.76 with p = 0.005. Segregating by Gleason score did not improve the AUC values for other biomarkers.

## Discussion

There are many approaches to measuring novel CaP biomarkers beyond traditional ELISA. One of the fundamental bottlenecks in most assay development schemes is the identification of a suitable matched antibody pair. This is perhaps the most important advantage of the bead based approach used in the current work—the identification of a matched antibody pair usually takes less than 2 days from the synthesis of the reagents to the final selection of the optimal pair. This system has good inter- and intra-assay reproducibility (CV<10%) and can quickly minimize matrix interferences. Other advantages include the lower detection limits because of avidity in the bead design as well as low threshold for user training. Limitations of the system include the relatively high sample volume requirements because of restricted multiplexing. To solve this, we are developing additional analytical tools in our labs such as the magneto-nanosensor for multiplexed analyses with large dynamic ranges and low sample volume (40 μl) requirements critical for precious samples [4].

It is important that biomarker verification be allowed to fail quickly before significant investments are made in test development or use of precious clinical samples. This bead-based scheme allows rapid verification of biomarker potential. Our data shows that many of the protein biomarkers reported previously to discriminate between CaP and BPH patients failed with our specific patient samples. Perhaps most surprisingly was CA1. In previous work using 54 CaP samples and 60 healthy controls [23], this protein had an AUC value of 0.64 and an AUC value of 0.76 when combined with PSA. One important difference is in that study restricted their samples to patients in the so-called “grey zone” of PSA testing—PSA values between 4 and 10 ng/mL. IL-6sr has also been shown to correlate with progression [27] and bone metastasis [26] and it was plausible that differential expression would have been measured in BPH versus CaP patients samples, however we noted no significant difference between these two patient populations.

Our work with SPON2 shows that it does have some utility in CaP testing. One curious feature from this study is that the levels in BPH patients (126.5 ng/mL) was actually higher than CaP patients (73.0 ng/mL). This difference was very significant at p = <10^−6^. In a previous paper [31], SPON2 levels in CaP patients (77.5 ng/mL) were higher than in patients with “no evidence of malignancy” (23.6 ng/mL). While the differences in absolute values are likely attributable to differences in standard and antibody selection, the trend is somewhat confusing. One possible explanation is in patient selection differences. These normal healthy controls are a much different patient cohort than the BPH group used for comparison in our study. Indeed, it is possible that SPON2 levels increase during BPH above values seen in either normal or CaP subjects. Future work will have to further study SPON2 levels in multiple patient and control groups with high numbers of patients to better understand these discrepancies.

Another study suggested that SPON2 is only applicable to patients with PSA values below 10 ng/mL [30]. Restricting our samples to these patients gave a mean BPH value of 119.9 ± 65.9, a mean CaP value of 82.6 ± 50.36 (p = 0.004) and AUC of 0.72. Thus, restricting our data to these patients did not improve the AUC.

Analysis of the post-surgery patients showed that the most dramatic changes in biomarker levels occurred in PSA-based tests. The tPSA in post-prostatectomy patients decreased 18-fold versus BPH and 30-fold for the CaP patients. PAP levels in the post-prostatectomy patients was 2- to 3-fold lower. Other biomarkers showed no significant (P>0.05) change.

## Conclusion

We report a piezoelectric bead-based sensor to measure serum proteins with potential to discriminate between BPH and CaP. This study reconfirmed the use of PSA to discriminate between BPH and CaP, but the AUC values were sub-optimal for population-wide screening. We also showed that using SPON2 in tandem with PSA via an OR operation can increase the AUC from 0.80 to 0.84. This approach has utility for a variety of circulating biomarkers and future work will evaluate more promising biomarkers as well as urine-based biomarkers such as the TMPRSS2:ERG fusion.

## Supporting Information

S1 Fig Optimization of Bead and d.Ab Concentration. Variations in d.Ab concentration two-fold above and below the recommended 200 ng/mL value were used in addition to increasing concentrations of beads (inset). Calibration curves at each of these points illustrate that the response is stable ± 15% despite these variations.
(PDF)

S1 Table Biomarker Concentrations as a Function of Gleason Score. PSA showed the most significant differences between patients with Gleason scores of 6 and 7.
(PDF)

S1 Video Mechanisms of Action of the Bead-based Analyzer. This video illustrates the assay parameters and shows how piezo-based signaling is translated to concentration of biomarker. Reprinted from BioScale Inc under a CC BY license, with permission from BioScale Inc, original copyright 2011.
(MP4)
