# Supplementary figures and images for: A Magnetic Bead-Based Sensor for the Quantification of Multiple Prostate Cancer Biomarkers
Source: PLoS One. 2015 Sep 30;10(9):e0139484. doi: 10.1371/journal.pone.0139484 (PMC4589536; doi:10.1371/journal.pone.0139484)

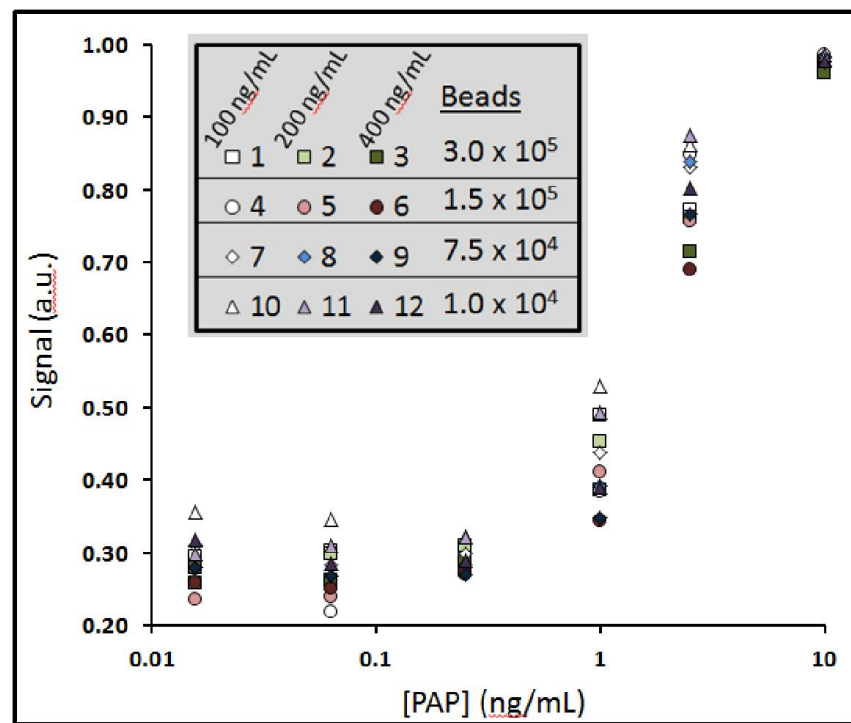

S1 Fig.

Supplement: S1 Fig — Variations in d.Ab concentration two-fold above and below the recommended 200 ng/mL value were used in addition to increasing concentrations of beads (inset). Calibration curves at each of these points illustrate that the response is stable ± 15% despite these variations. (PDF) [file pone.0139484.s001.pdf]
