# Supplementary material for: A Magnetic Bead-Based Sensor for the Quantification of Multiple Prostate Cancer Biomarkers
Source: PLoS One. 2015 Sep 30;10(9):e0139484. doi: 10.1371/journal.pone.0139484 (PMC4589536; doi:10.1371/journal.pone.0139484)
Supplement: S1 Table — PSA showed the most significant differences between patients with Gleason scores of 6 and 7. (PDF) [file pone.0139484.s002.pdf]

S1 Table.

**Biomarker**	**G6 (ng/mL)**	**G7 (ng/mL)**	**P value**
tPSA	12.9 ± 8.4	11.9 ± 13.1	<0.01
fPSA	0.79 ± 0.75	2.21 ± 0.67	0.58
PAP	2.0 ± 1.0	2.6 ± 1.2	0.11
SPARC	840 ± 334	1731 ± 294	0.96
CA1	1738 ± 2236	585 ± 1664	0.07
IL-6sR	75.3 ± 13.9	75.2 ± 22.3	0.09
SPON2	83 ± 52	243 ± 43	0.16
